# Human herpesvirus 6: An emerging pathogen.

**DOI:** 10.3201/eid0503.990306

**Published:** 1999

**Authors:** G Campadelli-Fiume, P Mirandola, L Menotti

**Affiliations:** University of Bologna, Italy. campadel@kaiser.alma.unibo.it

## Abstract

Infections with human herpesvirus 6 (HHV-6), a beta-herpesvirus of which two variant groups (A and B) are recognized, is very common, approaching 100% in seroprevalence. Primary infection with HHV-6B causes roseola infantum or exanthem subitum, a common childhood disease that resolves spontaneously. After primary infection, the virus replicates in the salivary glands and is shed in saliva, the recognized route of transmission for variant B strains; it remains latent in lymphocytes and monocytes and persists at low levels in cells and tissues. Not usually associated with disease in the immunocompetent, HHV-6 infection is a major cause of opportunistic viral infections in the immunosuppressed, typically AIDS patients and transplant recipients, in whom HHV-6 infection/reactivation may culminate in rejection of transplanted organs and death. Other opportunistic viruses, human cytomegalovirus and HHV-7, also infect or reactivate in persons at risk. Another disease whose pathogenesis may be correlated with HHV-6 is multiple sclerosis. Data in favor of and against the correlation are discussed.


### Human Herpesvirus 6: An Emerging Pathogen

**Gabriella Campadelli-Fiume, Prisco Mirandola, and Laura Menotti**
University of Bologna, Bologna, Italy

Infections with human herpesvirus 6 (HHV-6), a ß-herpesvirus of which two variant groups (A and B) are recognized, is very common, approaching 100% in seroprevalence. Primary infection with HHV-6B causes roseola infantum or exanthem subitum, a common childhood disease that resolves spontaneously. After primary infection, the virus replicates in the salivary glands and is shed in saliva, the recognized route of transmission for variant B strains; it remains latent in lymphocytes and monocytes and persists at low levels in cells and tissues. Not usually associated with disease in the immunocompetent, HHV-6 infection is a major cause of opportunistic viral infections in the immunosuppressed, typically AIDS patients and transplant recipients, in whom HHV-6 infection/reactivation may culminate in rejection of transplanted organs and death. Other opportunistic viruses, human cytomegalovirus and HHV-7, also infect or reactivate in persons at risk. Another disease whose pathogenesis may be correlated with HHV-6 is multiple sclerosis. Data in favor of and against the correlation are discussed.

### The Discovery of Human Herpesvirus 6 (HHV-6)

Initially designated HBLV, for human B-lymphotropic virus, HHV-6 was isolated fortuitously in 1986 from interleukin 2-stimulated peripheral blood mononuclear cells (PBMCs) of patients with AIDS or lymphoproliferative disorders (1). The PBMC cultures exhibited an unusual cytopathic effect characterized by enlarged balloonlike cells. The causative agent was identified as a herpesvirus by electron microscopy and lack of cross-hybridization to a number of human herpesviruses (2). The GS strain is the prototype of the first isolates. Two additional isolates of lymphotropic human herpesviruses, U1102 and Gambian, genetically similar to HBLV, were obtained 1 year later from PBMCs of African AIDS patients. All of the isolates could grow in T cells (CEM, H9, Jurkat), in monocytes (HL60, U937), in glial cells (HED), as well as in B-cell lines (Raji, RAMOS, L4, WHPT) (3,4). A new variant, Z29, subsequently shown to differ in restriction endonuclease pattern from GS-like strains, was isolated from PBMCs of patients with AIDS (5). The cells supporting virus growth were characterized as CD4+ T lymphocytes (6). The designation HHV-6 was proposed 1 year after discovery of the first isolate to comply with the rules established by the International Committee on Taxonomy of Viruses (7).

More than 100 additional HHV-6 strains have been isolated from PBMCs of children with subitum or febrile syndromes (8), from cell-free saliva of healthy or HIV-infected patients (9,10), from PBMCs of patients with chronic fatigue syndrome (CFS) (11), and from PBMCs of healthy adults—these PBMCs were cultivated for human herpesvirus 7 (HHV-7) isolation (12).

### The Virus

HHV-6 and HHV-7 belong to the *Roseolovirus* genus of the ß-herpesvirus subfamily; HHV-6 species are divided into two variants: HHV-6A and HHV-6B. The virion particle is 160 nm to 200 nm and has the morphologic features typical of herpes virion particles (a central core containing the viral DNA, a 90-nm to 110-nm capsid, and a tegument layer surrounded by a membrane structure) (13). We summarize briefly key features.

Address for correspondence: G. Campadelli-Fiume, Dipartimento di Patologia Sperimentale, Sezione di Microbiologia e Virologia, Via San Giacomo, 12, 40126 Bologna, Italy; fax: 39-051-354-747; e-mail: campadel@kaiser.alma.unibo.it.

HHV-6A genomes are 159 kbp to 170 kbp long. As sequenced, the genome of U1102 strain is 159 kbp long (14); the HHV-6B genome has been sequenced only partially. Seven gene blocks in the central region (I-VII) designated as herpesvirus core genes are common to all Herpesviridae. Another block, spanning open reading frames (ORFs) U2 to U14, contains genes specific to ß-herpesviruses. A further region, encompassing ORFs U15-U25, contains genes specific to *Roseolovirus* genus. Three genes (U22, U83, U94) are specific to HHV-6 and absent from HHV-7 (Figure 1). The closest homology and similarity in genome organization is to HHV-7 and next to human cytomegalovirus (HCMV). Amino acid similarity to HHV-7 is 46.6% to 84.9% and to HCMV 41.0% to 75.8% (14,16). The HHV-6 genome is composed of a unique sequence (85% to 90% of the genome) bracketed by direct repeats (10% to 15% of the genome) that contain the cleavage and packaging sequences *pac*-1 and *pac*-2 and a single origin of replication (OriLyt) located at 70 kbp of the genome. The number of predicted ORFs, 102 or 85, varies depending on the values used to define an ORF and was attributed mainly

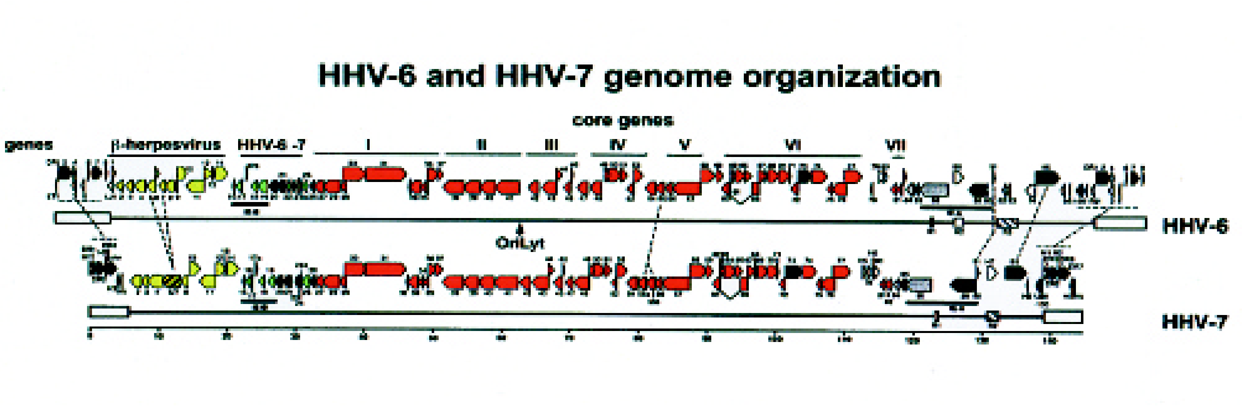

Figure 1. Schematic representation of HHV-6 and HHV-7 genomes. The genomes are colinear. Homologies are 46.6% to 84.9%. Red blocks represent the herpesvirus core genes, numbered from I to VII. Yellow blocks represent ß-herpesvirus subfamily-specific genes (from U2 to U14). Green blocks indicate genes present only in the *Roseolovirus* genus, i.e., in HHV-6 and HHV-7. Only three ORFs (U22, U83 and U94) are present in HHV-6 and absent from HHV-7 (modified from [15]).

on the basis of the similarity with HCMV (14) or HHV-7 (17) counterparts. Few gene products have been characterized so far. They include the immediate-early gene IE-A, which together with IE-B constitutes the IE locus, a highly spliced region with an arrangement similar to that of HCMV (18); the U3 gene, a homolog of the HCMV $U_L$24 gene, with transactivating activity (19); the origin binding protein (20), the U53 protease (21); and p100, also designated p101, highly immunogenic, and most probably a constituent of the tegument (22,23). In addition, HHV-6 (but not HHV-7) carries a homolog of the adenoassociated type 2 parvovirus *rep* gene (24), which is transcribed in latently infected cells (25). Recently, the U12 protein was recognized as a ß-chemokine receptor (26). A major focus has been in the glycoprotein field. Five glycoproteins were identified: gB (U39, gp116) (27-30), gH (U48, gp100) (31), and gL (U82, gp80), which form at least a heterodimer, gM (U72), and gp82-105 (U100) (29,30,32,33). gB and gH/gL were shown to be virion constituents, and antibody to gH can neutralize virion infectivity and syncytia formation, suggesting a role of gH in virus entry and in virus-induced cell fusion (31). The HHV-6 genome sequence predicts a locus of glycoproteins U20-U24 and U85 that are specific to the *Roseolovirus* genus (14), but the proteins have not yet been identified. U20 and U85 have a predicted immunoglobulin structure.

### Variant A and Variant B HHV-6 Strains

Frenkel and co-workers (34), Ablashi et al. (35), and Aubin et al. (36) were the first to discover that HHV-6 isolates display genetic and phenotypic variations. All the strains derived so far segregate into two groups, variant A and variant B, whose genome organization appears to be overlapping. Viruses belonging to the two variants differ with respect to several properties. Differences in restriction endonuclease cleavage sites are scattered throughout the entire genomes. Extent of homologies at nucleotide level varies from 99% to 95% for the most conserved genes located in the center of the genome to approximately 75% for the most divergent portions, located in the immediate-early region. Major differences in biologic properties concern the in vitro cell tropism, regulation of transcription and splicing patterns, reactivity to some MAbs directed to variant-specific epitopes (29,34,35). Typically, variant A viruses replicate in HSB-2 cells, whereas the variant B viruses grow in the less differentiated Molt3 T-cell lines. Variant B viruses grow to higher yields than variant A viruses in primary human fetal astrocytes and require IL-2- and phytohemagglutinin-activated PBMCs. Differences between the two variants affect the regulation of transcription of some ORFs of the immediate early region-B and-A (U16, U17, U91) and the splicing pattern of ORFs U18-U20 (37). The differences relative to infection in humans (epidemiology, correlation with pathologic features, tissue tropism) are detailed below and in the table.

All strains fall into one or the other variant group. There is no evidence of recombination or a genetic gradient, which suggests that in vivo the two groups of viruses occupy different ecologic niches. Any isolate characterized for more than one marker has been unambiguously assigned to one or the other variant group. The designation of the two groups as variants has been highly debated and controversial (38). A key question is whether the two variants fulfill the criteria defined by the International Committee on Taxonomy of Viruses for classification as different species (13). In our opinion, the information summarized above indicates that the two variant groups may be different species; therefore, the issue of nomenclature should be reconsidered.

Table. Epidemiology and distribution of human herpesvirus (HHV-6) variants

| | Variant A | Variant B |
|---|---|---|
| **Associated pathologic conditions** | | |
| Exanthem subitum, febrile syndromes[b] | –[a] | ++++ |
| Multiple sclerosis | ++ | ++ |
| Lymphomas and neoplasies | ++ | ++ |
| Reactivation in transplants | ++ | ++ |
| Reactivation in AIDS | ++ | ++ |
| **Tissue distribution** | | |
| Peripheral blood mononuclear cells | + | +++ |
| Salivary glands | – | ++++ |
| Skin | ++ | ++ |
| Brain | ++ | ++ |
| Lymph nodes | – | ++++ |
| Other tissues | – | ++++ |
| Serum from healthy persons | – | – |
| Serum from exanthem subitum patients | – | ++++ |
| Serum from other patients[c] | +++ | + |
| Saliva | – | ++++ |
| Cerebrospinal fluid | +++ | + |

[a]Different degrees of HHV-6 positivity.
[b]Exception, Zambian children, 44% variant A.
[c]Patients with AIDS, chronic fatigue syndrome, and lymphomas.

### Natural History of HHV-6 Infection

Infection with HHV-6 is very common, approaching 100% in seroprevalence. Exceptions, if confirmed, are represented by countries (e.g., Morocco) where seroprevalence is much lower (20%) (39). Antibody titers are high in newborn children, drop at 3 to 9 months after birth, rise again briefly thereafter, and remain elevated until the age of 60 or older. This pattern indicates that newborns carry maternal antibodies and primary infection occurs in the first 3 years of life, most frequently the first year. Transplacental infections are very infrequent but may contribute to HHV-6 seropositivity in newborns (40).

Three stages can be recognized in the natural history of HHV-6 infection (Figure 2). The first is represented by acute primary infection in infants. The second occurs in healthy children and adults; the virus replicates in the salivary glands and is secreted in saliva (for HHV-6B) without inducing any obvious pathology, remains latent at least in lymphocytes and monocytes, and persists in various tissues, possibly with a low-level replication. The third stage occurs infrequently, typically in immunocompromised persons, and is linked to reactivation of virus from latency or reinfection.

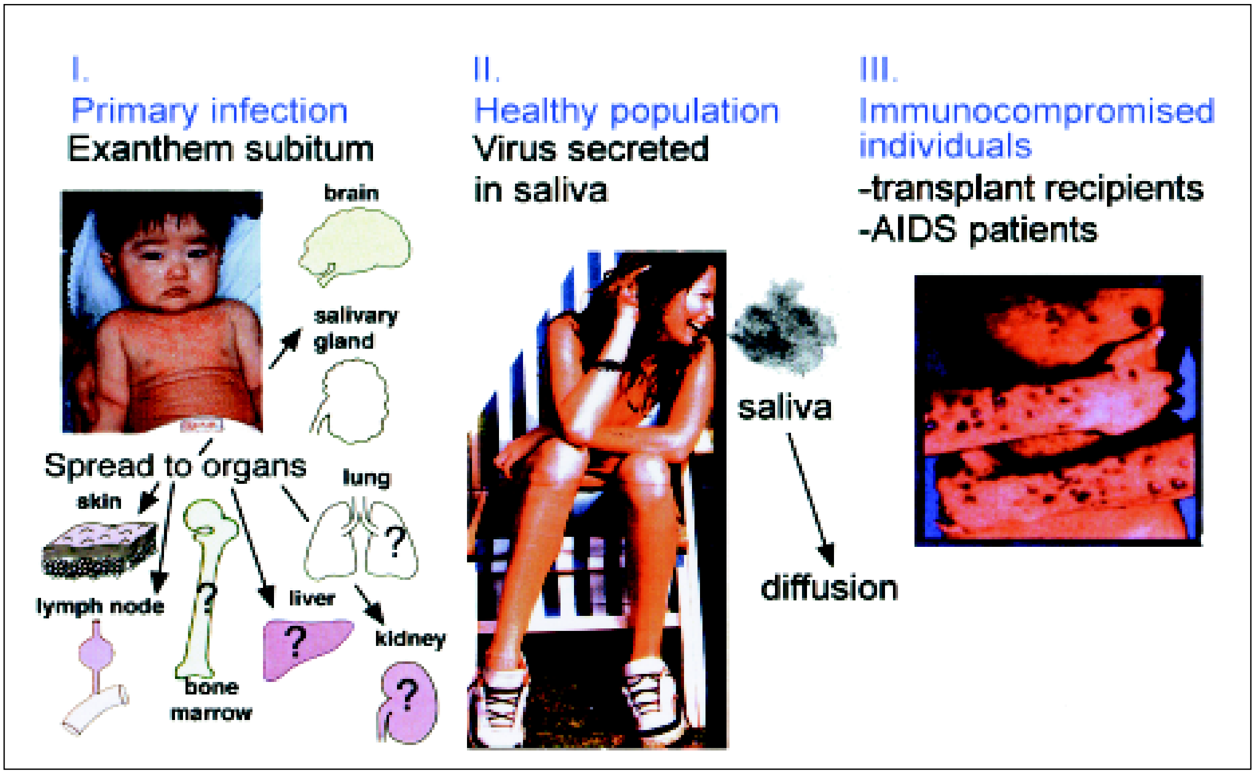

Figure 2. Stages of the natural history of HHV-6 infection: I. Primary infection occurs in infants, may result in exanthem subitum (rash on the child's chest), and spreads to organs. Question marks denote sites where HHV-6 spread is likely but not proven. II. In healthy infants and adults, HHV-6 is present in a latent or persistent form in lymph nodes and is produced asymptomatically in salivary glands and shed in saliva, the most probable route of transmission. III. HHV-6 infection/reactivation occurs in persons undergoing therapeutic immunosuppression after organ transplant or in AIDS patients.

Other pathologic conditions, mainly multiple sclerosis, tumors, and CFS have been linked to HHV-6.

### Primary Infection

The unequivocal demonstration that primary infection with HHV-6B causes roseola infantum was provided by Yamanishi et al. (8), who investigated the correlation between seroconversion to HHV-6B and childhood infectious diseases and found that seroconversion occurs concomitantly with roseola infantum, also designated exanthem subitum or sixth disease, a common, mild, acute febrile disease of infants. Fever lasts for a few days and is sometimes followed by a maculopapular rash that resolves spontaneously. Primary infection may be asymptomatic or may cause clinical manifestations other than classic exanthem subitum. In four studies, children admitted to emergency clinics with febrile illnesses were HHV-6-positive in approximately 10% to 15% of cases and in one study in approximately 45% of cases, as determined by viral isolation, seroconversion, or detection of viral DNA sequences in PBMCs. Other than rash, symptoms included otitis, gastrointestinal or respiratory distress, and seizures (41-44). Complications of primary HHV-6 infections are uncommon and rarely fatal; they were described mainly as case reports and include invasion of the central nervous system (CNS) with seizures, hyperpyrexia, vomiting, diarrhea, cough, emophagocytic syndrome, fulminant hepatitis, disseminated infection, and hepatosplenomegaly. These complications suggest that the virus may spread to a number of organs, which may represent potential sites of virus persistence or latency and (subsequently) reactivation. For example, seizures and other CNS complications are clear indications of invasion of this organ and correlate well with neurotropism of HHV-6.

HHV-6 primary infection accounts for 10% to 45% of cases in children admitted to emergency clinics with febrile illness and 1% of cases in hospitalized children (42).

HHV-6B is not the only causative agent of exanthem subitum. Occasionally, HHV-7 may also cause fever with or without rash. Primary infection with HHV-7 occurs at a somewhat later age than with HHV-6B. Initially, it was proposed that pathologic manifestations seen during primary HHV-7 infection were the consequence of HHV-6 reactivation by HHV-7. Evidence that HHV-7 by itself causes an exanthematic disease, although less frequently than HHV-6B, rests on the finding that children with exanthem subitum seroconvert to HHV-7 but remain HHV-6B-negative (45,46).

Virus replicated in the salivary glands and secreted in saliva is the epidemiologically proven source of transmission. Other routes of transmission have been suggested but remain to be proven. HHV-6B DNA was recovered from cervical tissues and secretions (47-49), but children born to mothers with positive cervical swabs did not acquire the infection. Intrauterine transmission was suggested by polymerase chain reaction (PCR) positivity of uncultured cord blood mononuclear cells (CBMCs) in 1.6% of the cases and by a case of abortion of an HHV-6-positive fetus (40). Transmission through breastfeeding is also doubtful since HHV-6 DNA, unlike HHV-7, is not found in breast milk (50). Integration of the HHV-6 genome in lymphoblasts from a leukemic patient and his offspring raised the possibility of genetic transmission. As vertical transmission was not observed in other cases of genome integration, the presence of HHV-6 DNA in offspring was alternatively interpreted as a tendency of HHV-6 to integrate at specific chromosomal loci (51,52).

With the exception of a strong association of HHV-6A with febrile syndromes in Zambian children (43), which could reflect an endemic variant A hot spot, HHV-6A has rarely been isolated or detected in children with primary HHV-6 infection (53,54). The age at which primary HHV-6A infection occurs and the diseases clearly linked to it have not been determined.

### HHV-6 in Healthy Persons

The second stage of HHV-6 infection occurs in healthy children and adults, in whom the virus actively replicates in the salivary glands, is latent in at least lymphocytes and monocytes, and persists in various tissues. Replication in salivary glands—observed for HHV-6B but not HHV-6A (9,10,47)—accounts for the route of transmission and for the high frequency of detection and isolation of virus in saliva. Lymphocytes, and probably monocytes, represent two known sites of latency, as the virus can be reactivated from PBMCs and adherent monocytes upon cultivation (55), and viral DNA sequences are detected in PBMCs of as much as 90% of the population. Additional sites of latency likely exist since the virus or viral sequences can be readily detected in a number of tissues. A form of latent infection is represented by integration of the HHV-6 genome in the host chromosomes (51,52). Persistence of HHV-6 in cells and tissues is discussed in the section In Vivo Tropism.

A missing link in our understanding of the natural history of HHV-6 infection is the source of the virus that spreads to organs. Monocytes have a short half-life; they may be vehicles of virus spread to organs, but they themselves need to be infected. A possible source may be virus produced in the salivary glands. In one case, early bone marrow progenitor cells were found to be latently infected in vivo (56), which raises the possibility that they may represent a site of latency, and by corollary, upon viral reactivation from latency, an alternative source by which virus spreads to tissues.

In immunocompetent adults, infection or reactivation of HHV-6 at sites other than the salivary glands is rare. Occasionally, infection results in lymphoadenopathy, fulminant hepatitis, mononucleosislike syndrome, or generalized infection.

### HHV-6 in the Immunosuppressed

The third stage of HHV-6 infection, which occurs in the immunosuppressed, is responsible for the most serious clinical manifestations associated with HHV-6 infection or reactivation. Persons at risk are recipients of bone marrow, kidney, and liver transplants, in whom immunosuppression is induced for therapeutic reasons. In these patients, HHV-6 infection or reactivation may result in bone marrow suppression, pneumonitis, encephalitis, encephalopathy, hepatitis, fever, and skin rash or may complicate engraftment of the transplanted organ and culminate in rejection and death. As the number of persons undergoing organ transplantation and, consequently, subjected to therapeutic immunosuppression increases, the number of persons at risk is increasing. Assessment of the contribution of HHV-6 to posttransplant complications is made more difficult by the presence of other opportunistic viruses and by the scarcity of thorough studies on all the viruses present in these organs. Thus, most of the reports on the presence of HHV-6 did not deal with the fact that HCMV reactivation is frequent in transplant recipients (particularly kidney) and may occur together with HHV-6 reactivation. When investigated in detail, a synergistic effect of HHV-6 and HCMV was apparent in renal transplant recipients, and the simultaneous detection of both HHV-6 and HCMV DNAs in urine or serum or of immunoglobulin (Ig) M antibodies was the strongest predictor of viral disease and of severity of disease (57,58). HHV-7 can also reactivate in transplant recipients (59), again alone and in association with HHV-6 or HCMV. Each of these viruses is a pathogen in its own right, and in combination with the other, may produce disease far more serious in outcome and clinical manifestations than it would alone. In many studies, no effort was made to identify the HHV-6 variant. When the variants were characterized, a rather heterogeneous pattern emerged. In PBMCs, brain and lungs variant B strains were predominant (60-62), whereas in spinal fluid and serum, variant A strains were prevalent (63,64). In approximately 30% of bone marrow transplant recipients in whom pneumonitis developed, both variants were simultaneously detected (62), an otherwise rare occurrence.

AIDS patients are the second group of immunocompromised persons at risk for HHV-6 and HCMV-related opportunistic viral infections. The overall incidence of these infections has decreased substantially after the introduction of highly active antiretroviral therapy. HHV-6 infection/reactivation in AIDS patients results in an increase in HHV-6 load both in lymph nodes and generalized, in viremia, disseminated infection in many organs, active CNS infection, pneumonitis, and retinitis and may contribute as a cause of death (65-67). These findings lead to the proposal that HHV-6 acts as a cofactor in the progression of AIDS and in the switch of HIV from the latent to the replicative state (68). Although a significant increase in HHV-6 viral load was not observed in PBMCs of HIV-seropositive persons (68,69), HHV-6 and HIV could interact in lymph nodes. The possibility that HHV-6 acts as a cofactor in AIDS progression boosted intense research on mutual interactions between HHV-6 and HIV in cell cultures and cell-free systems. In addition to coinfection, observed in vivo and in vitro, HHV-6 promotes HIV replication through upregulation of cytokines (e.g., TNF-α and IL-1ß) and through transactivation of the long terminal repeat by IE-A and IE-B (68). The possibility that in vivo HHV-6 infection may lead to HIV reactivation was examined recently in HIV-positive children. The children in whom AIDS progressed rapidly acquired primary HHV-6 infection later than those in whom HIV progressed slowly; however, in the rapid progress to AIDS the HIV viral load did not increase at the outcome of HHV-6 infection. Late in AIDS, HHV-6 detection in PBMCs is reduced, most likely because of T-cell depletion (69). As a rule, the variant A strains are more frequently associated with AIDS patients.

### In Vivo Tissue Distribution

In addition to the salivary glands, HHV-6 has been frequently isolated from cultured PBMCs from AIDS patients or children with exanthem subitum or febrile illnesses. This led to the initial definition of HHV-6 as a lymphotropic virus. In lymphocytes, the virus establishes a latent infection, readily monitored by PCR amplification of viral DNA sequences in uncultured PBMCs (47). Furthermore, productive infection has been monitored in some cases by immunohistochemistry (e.g., in CD4+ T lymphocytes) (70). In contrast with the earlier view of HHV-6 as a T-lymphotropic virus, recent investigations detected HHV-6 in many tissues. Despite the wealth of research on the presence of HHV-6 in humans, our knowledge is fragmentary. By immunohistochemical staining, active HHV-6 infection was detected in various cells (e.g., CD68+ cells of the monocyte/macrophage lineage in Kaposi sarcoma [71], epithelial cells and lung macrophages, dendritic cells and macrophages of lymph nodes and infiltrating lymphocytes of organs of unselected patients who died of AIDS, tubular epithelial cells of kidney) and in submandibular glands (67,72). Consistent with this wider host range, HHV-6 DNA sequences were detected in a number of organs (e.g., skin, spleen, lung, heart, kidney, adrenal gland, esophagus, duodenum, colon, liver, and early bone marrow progenitor cells) from patients who died of heart attack or accidents (47,56,65). Since in numerous studies detection was performed by PCR, latent, persistent, or productive infections were not differentiated, nor was the nature of the infected cells defined. Variant B strains are more frequently found in both PBMCs and solid tissues. Variant A viruses appear predominant in skin and can replicate in primary fibroblast cultures, suggesting a preferential tropism for skin (47). HHV-6 is a brain commensal (see section entitled Neurotropism and Multiple Sclerosis).

### In Vitro Tropism

In vitro, HHV-6 infects and replicates at highest titers in PBMCs and CBMCs. In these heterogeneous cultures, susceptible cells are the CD4+ T lymphocytes but also the CD4-CD3+CD8+ and the CD4-CD3- natural killer cells (68). Inasmuch as CD4 expression is not a requirement for susceptibility to HHV-6 infection and soluble forms of CD4 and antibodies to CD4 fail to inhibit virus infectivity (73), CD4 is not a necessary component of the cellular receptor for HHV-6. In addition to primary T lymphocytes, T-lymphocytic lines (e.g., HSB-2, SupT1, Molt3, JJhan, MT-4, ET62) support HHV-6 growth. Viruses of the two variants display different host range, as variant A strains generally do not replicate in Molt3 cells, whereas variant B strains do not replicate in HSB-2 cells. Permissive cells of lineages other than T lymphocytes are the liver cell line HepG2 (74) and a number of human and nonhuman cell lines in which the virus generally grows at very low yields (e.g., cervical cells lines, human primary astrocytes [B variant does not replicate very well] neuroblastoma, human bowel-derived cell monocytes, megakaryocytes endothelial cells, NBL-7 mink lung epithelial cells, and PBMCs of several Macaca species) (75-77). Altogether, in cell cultures as well as in vivo, HHV-6 appears to have a host range wider than initially recognized, extending beyond T lymphocytes. While this is meaningful with respect to studies on the natural history of the infection, the practical use of these cells in the laboratory is hampered by the very low virus yields. Even in the most permissive systems (PBMCs, CBMCs, and T-cell lines), the virus yields are very low. In our experience, CBMC cultures, the most productive cell type, do not yield more that $10^4$ infectious units per ml, whereas the titer of a herpes simplex virus type 1 stock is generally as high as $10^9$- $10^{10}$ plaque-forming units per ml.

### Neurotropism and Multiple Sclerosis

HHV-6 is probably the most neurotropic virus known. Neuroinvasion has been documented in infants with primary infection, in focal encephalitis, in children and adults with AIDS, in recipients of bone marrow transplants, as well as in immunologically competent children and adults. Challoner et al. (78) reported viral DNA sequences in approximately two thirds of brain specimens and viral antigen expression in a number of cell types (e.g., astrocytes, macrophages, epithelial cells, endothelial cells of blood vessels) at very similar frequencies in specimens from healthy persons and multiple sclerosis patients. Astrocytes were confirmed as a susceptible cell population, although in a subsequent study only samples from AIDS patients were positive (79).

Both variant viruses were detected in the brain of patients who died of causes related or unrelated to HHV-6, which demonstrates that both variant viruses can invade and be harbored in the brain (61,78-82). Although studies on the differential distribution of the two variant groups provided conflicting results (78,83), for HHV-6B, CNS invasion has been documented at primary infection. Instead, for HHV-6A, the time of CNS invasion has not been documented.

A possible correlation between active HHV-6 infection and multiple sclerosis has been the focus of much attention in the past few years. Multiple sclerosis is a severe CNS disease of young adults, characterized by the progressive demyelination of nerves that leads to progressive paralysis and eventually death. The disease appears to be an autoimmune reaction to myelin, the coating of nerve fibers. Viruses have long been suggested as etiologic agents of myelopathies, and DNA sequences from a number of viruses, particularly herpesviruses, have been detected, although not consistently. In addition, since multiple sclerosis is accompanied by a characteristic increase in IgG titer in serum and spinal fluid, antibodies to various viruses (including HHV-6) have been frequently searched for. Even immunologic studies have been inconclusive, most probably because the increase in antibody response reflects an immune dysfunction or different genetic background together with damage of the blood-brain barrier, rather than an epidemiologic correlation with any given virus.

Studies of HHV-6 infection or reactivation in multiple sclerosis patients have provided controversial results. In initial reports, active infection was suggested by an increase in IgG titer in both serum and spinal fluid but was not confirmed by increase in PCR positivity of PBMCs (84). By representational difference PCR, Challoner et al. (78) found that multiple sclerosis specimens contained HHV-6B DNA sequences. HHV-6B antigen expression was detected at higher frequency in multiple sclerosis plaques than in histologically normal specimens. Nuclei of oligodendrocytes were positive in multiple sclerosis samples (80%) but not in control samples (0%) and were interpreted as a hallmark of the association between active or reactivable HHV-6 infection and the disease. Attempts to reproduce the immunohistochemical results were not successful, and viral expression as documented by reverse transcriptase (RT)-PCR was also negative (85). In favor of a correlation are subsequent findings that the frequency and titer of anti-HHV-6 IgM antibodies are higher in samples from multiple sclerosis patients than from controls (73% vs. 18%) and that the serum DNAemia was specifically positive in multiple sclerosis patients (30% vs. 0%) (86). HHV-6 DNA sequences had been detected in spinal fluid, but not in serum from multiple sclerosis patients (87). Critical interpretation of these data can be summarized as follows. The serologic analyses are difficult to interpret as this disease is characterized by an immunologic dysregulation; therefore, the increase in antibody titer may be a sign of the disease rather than a cause. The PCR data were not confirmed. Thus, no statistical difference was reported in DNA positivity of plaques (32% active vs. 17% inactive plaques) (88), no DNA was detected in serum and cerebrospinal fluid samples (89-91), and no viral RNA was found by RT-PCR in multiple sclerosis brain specimens (85). The differences in PCR results may reflect differences in PCR conditions (e.g., primers, number of cycles, characteristics of the amplified sequences, nature, and conservation of the specimens analyzed) but do not account for the observed discrepancies. Therefore, correlation between active HHV-6 infection and multiple sclerosis is still a controversial issue rather than a firmly established conclusion.

### Kaposi Sarcoma

Kaposi sarcoma is a multifocal angioproliferative disease localized predominantly in the skin or mucous membranes and in other visceral organs and lymph nodes. In addition to the classic, iatrogenic, and endemic forms, the disease occurs frequently and aggressively in AIDS patients. Human herpesvirus 8 (HHV-8) sequences were detected for the first time in Kaposi sarcoma specimens (92,93) by representational difference analysis PCR; HHV-8 is being investigated as the possible etiologic agent. Epidemiologic studies had long suggested a viral etiology, and many viruses, including HHV-6 and HHV-7, were detected in Kaposi sarcoma tissues. While neither HHV-6 nor HHV-7 appears to contribute to its etiology, Kaposi sarcoma represents a unique and interesting environment for these viruses, and they may have a role in the progression of the tumor. By immunohistochemistry, HHV-6B has been localized to CD68+ cells of the monocytic macrophage lineage. These cells are either singly infected with HHV-6 or HHV-7 or doubly infected with HHV-6 and HHV-7 (Figure 3) (71). Although some tissues harbor both viruses, albeit in different cells (e.g., lungs and salivary glands), cells doubly infected with HHV-6 and HHV-7 have not been detected in any tissue other than Kaposi sarcoma lesions (94). In addition, in the case of HHV-7, CD68+ cells are a cell type infected, singly or doubly, in no other tissue but in this tumor (71). Data suggest that the particular microenvironment of Kaposi sarcoma lesions, which is rich in chemokines and cytokines, attracts circulating lymphocytes and monocytes that harbor HHV-6 and HHV-7 in a latent or persistent form, induces viral reactivation, and promotes viral growth. In this peculiar environment two unusual situations occur. Viral yields are high for both HHV-6 and HHV-7. This accounts for the likelihood of double HHV-6 and HHV-7 infection, which most likely appears to take place in the tumor itself. HHV-7 tropism is also not restricted to T lymphocytes and extends to CD68+ cells, a lineage not susceptible to HHV-7 infection in other tissues. A predicted chemokine (U83) encoded by HHV-6

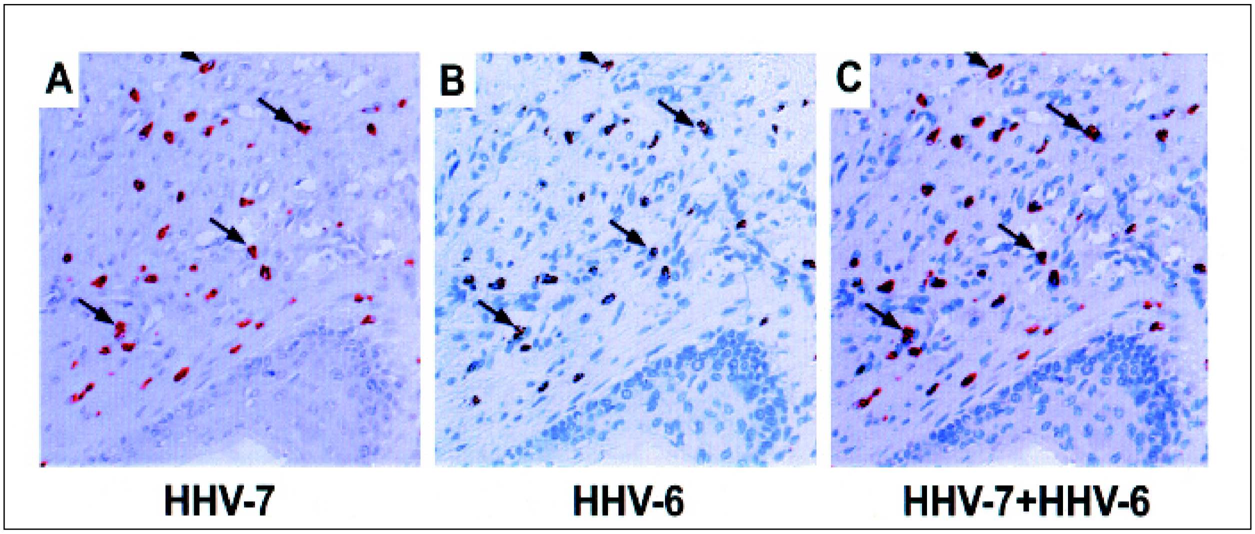

Figure 3. Expression of human herpesvirus 6B (HHV-6B) and HHV-7 antigens in serial sections of Kaposi sarcoma specimens. Panels A-C: In Kaposi sarcoma environment, cells can be doubly infected by HHV-6B and HHV-7: (A) Staining with monoclonal antibody 5E1 to HHV-7-specific antigen pp85; (B) Staining with monoclonal antibody to HHV-6B-specific antigen p101; (C) Overlaid serial sections show colocalization of HHV-6B and HHV-7 (71).

may contribute to dysregulating cellular chemokine expression or signaling. In addition, the virus expresses its own chemokine receptors encoded by the U12, and possibly U51 genes. Once HHV-6 is reactivated and actively replicating, HHV-6 may play a role in tumor progression through these molecules and mechanisms. Different studies detected different variant strains in Kaposi sarcoma tumors (95,96). The reason for this discrepancy is unknown.

### Lymphoproliferative and Neoplastic Disorders

Initial isolation of HHV-6 from patients with lymphoproliferative disorders boosted studies on possible association of HHV-6 with proliferative diseases, particularly of lymphoid origin, aimed at showing either a transforming potential of the virus in cell cultures or epidemiologic and molecular relationships between HHV-6 and various types of neoplasia.

In favor of a possible oncogenic potential is the transforming ability of three viral DNA fragments on mouse fibroblast cell lines or human epidermal keratinocytes. One encodes DR7 (97) and the other two encompass the regions spanning U2-U20 and U31-U37. The derived cellular clones were malignant and tumorigenic in athymic mice (98,99). DR7 and the two other loci also contain genes with transactivating activity on the HIV LTR.

Clinical and molecular investigations dealing with HHV-6 and various types of tumors have been reviewed (13). The overall importance of these findings remains controversial, mainly because the criteria for establishing an association between the virus and its oncogenic potential have not been met. Thus, the viral DNA sequences found in a tumor are expected to be the same as those with in vitro transforming potential, and in vitro-transformed cells should be tumorigenic in animals. In a given type of tumor the viral sequences should constantly be the same. In vitro-transformed cells and tumors should express the same viral gene products. The oncogenic potential of the virus should be demonstrated in a suitable animal model (which is lacking for HHV-6). Chromosomal integration of HHV-6 DNA in cells from lymphomas (51,52) may open a new scenario.

### CFS

CFS is an illness characterized by memory and attention impairment, muscle and multijoint pain, and unrefreshing sleep and weakness lasting longer than 6 months. The etiology of the disease is unknown, and many viruses have been investigated as possible causing agents. The overall scenario is in a way similar to that of multiple sclerosis. Serologic analysis on the presence of antibody to HHV-6 provided inconclusive data. An increase in IgG and IgM

titer in the sera of a large number of CFS patients (119 of 154) was found relative to that in the control population (77% vs. 12%) (100). However, this was not specific, as an increase in antibodies to other viruses was also detected, reflecting probably an immunologic dysfunction. Molecular analysis showed a higher prevalence of HHV-6A but not HHV-6B or HHV-7 in CFS patients (64,101,102), and HHV-6A could also be isolated from these patients (103). Whether this reflects an association or the consequence of an immune dysregulation remains to be determined.

### Conclusions

The epidemiologic and clinical investigations summarized here establish a clear correlation between HHV-6B primary infection and exanthem subitum and between HHV-6 infection/reactivation and a number of pathologic conditions in immunocompromised patients and transplant recipients. A firm correlation with other diseases remains doubtful. In the case of multiple sclerosis a clearly established correlation may identify patients who might benefit from specific anti-HHV-6 chemotherapy.

Yet another area deserving attention is the state of the virus in healthy people, a key prerequisite to understanding virus behavior in pathologic conditions. We have underlined that, in addition to establishing the true latent infection recognized in earlier studies, HHV-6 persists in the host through a combination of low-level persistent infection of various cells and tissues, a situation similar to that reported recently for HHV-7 (94). Sites of latency may represent a reservoir of the virus, which upon reactivation may feed the sites of persistency.

The pathogenic mechanisms of HHV-6 at the molecular, cellular, and tissue level remain largely obscure. Almost 10 years elapsed between the first isolation of HHV-6 and publication of the sequence of the entire genome. Now, single gene products can be studied in the context of the viral genome and in heterologous expression systems. Although a system for mutagenesis of the viral genome has yet to be developed, the stage is set to ask questions on the molecular mechanisms underlying pathogenicity of the virus. The forthcoming area of research will probably focus on links between the function of single gene products and mechanisms of pathogenesis and virus spread. A key feature of the HHV-6 life-style in the human host is its ability to infect and survive—in a latent or persistent form—in the cells of the immune system, and the pathogenic potential of HHV-6 is linked to its ability to evade immune system control. Analysis of the genomic sequence shows candidates for immune evasion strategies. Yamanishi and colleagues reported that a 7-transmembrane protein encoded by U12 acts as a ß-chemokine receptor (26). As ß-chemokines are key mediators of the immune response, the ß-chemokine receptor may subtract these mediators in a particular microenvironment. The immune evasion strategy must be more complex, as analysis of the viral DNA sequence shows additional candidates, e.g., a predicted chemokine encoded by ORF U83 and a second 7-transmembrane protein—a structural feature typical of chemokine receptors—encoded by ORF U51. This latter protein has a very unusual cell-type-dependent trafficking property (as it is transported to the plasma membrane in infected as well as transfected T lymphocytes) but fails to be transported to the plasma membrane in transfected human monolayer cells (104), raising the possibility that its function is regulated in a cell-dependent fashion through modulation of cell surface expression. Also U51 appears to dysregulate cellular chemokine expression (105). Studies of single gene products will probably lead to the identification of immunodominant proteins and the development of standardized recombinant diagnostic reagents.

The work done in our laboratory was supported by grants from AIDS Project from Istituto Superiore di Sanità, BIOMED2 BMH4 CT95 1016 grant from UE, Target Project in Biotechnology, MURST 40%, University of Bologna 60% and pluriannual plan.

Dr. Campadelli-Fiume is professor of microbiology, University of Bologna. Her research interests focus on human herpesviruses 6 and 7; identification, mapping, and expression studies of the major viral glycoproteins and immunodominant tegument proteins; and definition of the natural history of the infections. Her research on herpes simplex virus 1 deals primarily with mechanisms of virus entry into the cells and identification of cellular receptors for entry and the mechanisms of HSV transport and exit through the exocytic pathway.

### References

1. Salahuddin SZ, Ablashi DV, Markham PD, Josephs SF, Sturzenegger S, Kaplan M, et al. Isolation of a new virus, HBLV, in patients with lymphoproliferative disorders. Science 1986;234:596-601.
2. Josephs SF, Salahuddin SZ, Ablashi DV, Schachter F, Wong Staal F, Gallo RC. Genomic analysis of the human B-lymphotropic virus (HBLV). Science 1986;234:601-3.
3. Downing RG, Sewankambo N, Serwadda D, Honess R, Crawford D, Jarrett R, et al. Isolation of human lymphotropic herpesviruses from Uganda. Lancet 1987;2:390.
4. Tedder RS, Briggs M, Cameron CH, Honess R, Robertson D, Whittle H. A novel lymphotropic herpesvirus. Lancet 1987;2:390-2.
5. Lopez C, Pellett P, Stewart J, Goldsmith C, Sanderlin K, Black J, et al. Characteristics of human herpesvirus-6. J Infect Dis 1988;157:1271-3.
6. Takahashi K, Sonoda S, Higashi K, Kondo T, Takahashi H, Takahashi M, et al. Predominant CD4 T-lymphocyte tropism of human herpesvirus 6-related virus. J Virol 1989;63:3161-3.
7. Ablashi DV, Salahuddin SZ, Josephs SF, Imam F, Lusso P, Gallo RC, et al. HBLV (or HHV-6) in human cell lines. Nature 1987;329:207.
8. Yamanishi K, Okuno T, Shiraki K, Takahashi M, Kondo T, Asano Y, et al. Identification of human herpesvirus-6 as a causal agent for exanthem subitum. Lancet 1988;1:1065-7.
9. Levy JA, Ferro F, Greenspan D, Lennette ET. Frequent isolation of HHV-6 from saliva and high seroprevalence of the virus in the population. Lancet 1990;335:1047-50.
10. Harnett GB, Farr TJ, Pietroboni GR, Bucens MR. Frequent shedding of human herpesvirus 6 in saliva. J Med Virol 1990;30:128-30.
11. Ablashi DV, Lusso P, Hung CL, Salahuddin SZ, Josephs SF, Llana T, et al. Utilization of human hematopoietic cell lines for the propagation and characterization of HBLV (human herpesvirus 6). Int J Cancer 1988;42:787-91.
12. Katsafanas GC, Schirmer EC, Wyatt LS, Frenkel N. In vitro activation of human herpesviruses 6 and 7 from latency. Proc Natl Acad Sci U S A 1996;93:9788-92.
13. Braun DK, Dominguez G, Pellett PE. Human herpesvirus 6. Clin Microbiol Rev 1997;10:521-67.
14. Gompels UA, Nicholas J, Lawrence G, Jones M, Thomson BJ, Martin ME, et al. The DNA sequence of human herpesvirus-6: structure, coding content, and genome evolution. Virology 1995;209:29-51.
15. Nicholas J. Determination and analysis of the complete nucleotide sequence of human herpesvirus 7. J Virol 1996;70:5975-89.
16. Nicholas J, Martin ME. Nucleotide sequence analysis of a 38.5-kilobase-pair region of the genome of human herpesvirus 6 encoding human cytomegalovirus immediate-early gene homologs and transactivating functions. J Virol 1994;68:597-610.
17. Megaw AG, Rapaport D, Avidor B, Frenkel N, Davison AJ. The DNA sequence of the RK strain of human herpesvirus 7. Virology 1998;244:119-32.
18. Takeda K, Nakagawa N, Yamamoto T, Inagi R, Kawanishi K, Isegawa Y, et al. Prokaryotic expression of an immediate-early gene of human herpesvirus 6 and analysis of its viral antigen expression in human cells. Virus Res 1996;41:193-200.
19. Mori Y, Yagi H, Shimamoto T, Isegawa Y, Sunagawa T, Inagi R, et al. Analysis of human herpesvirus 6 U3 gene, which is a positional homolog of human cytomegalovirus UL 24 gene. Virology 1998;249:129-39.
20. Inoue N, Dambaugh TR, Rapp JC, Pellett PE. Alphaherpesvirus origin-binding protein homolog encoded by human herpesvirus 6B, a betaherpesvirus, binds to nucleotide sequences that are similar to ori regions of alphaherpesviruses. J Virol 1994;68:4126-36.
21. Tigue NJ, Matharu PJ, Roberts NA, Mills JS, Kay J, Jupp R. Cloning, expression and characterization of the proteinase from human herpesvirus 6. J Virol 1996;70:4136-41.
22. Neipel F, Ellinger K, Fleckenstein B. Gene for the major antigenic structural protein (p100) of human herpesvirus 6. J Virol 1992;66:3918-24.
23. Yamamoto M, Black JB, Stewart JA, Lopez C, Pellett PE. Identification of a nucleocapsid protein as a specific serological marker of human herpesvirus 6 infection. J Clin Microbiol 1990;28:1957-62.
24. Thomson BJ, Weindler FW, Gray D, Schwaab V, Heilbronn R. Human herpesvirus 6 (HHV-6) is a helper virus for adeno-associated virus type 2 (AAV-2) and the AAV-2 rep gene homologue in HHV-6 can mediate AAV-2 DNA replication and regulate gene expression. Virology 1994;204:304-11.
25. Rotola A, Ravaioli T, Gonelli A, Sgarzani C, Cassai E, Di Luca D. U94 of human herpesvirus 6 is expressed in latently infected peripheral blood mononuclear cells and blocks viral gene expression in transformed lymphocytes in culture. Proc Natl Acad U S A 1998;95:13911-6.
26. Isegawa Y, Ping Z, Nakano K, Sugimoto N, Yamanishi K. Human herpesvirus 6 open reading frame U12 encodes a functional beta-chemokine receptor. J Virol 1998;72:6104-12.
27. Foà-Tomasi L, Guerrini S, Huang T, Campadelli-Fiume G. Characterization of human herpesvirus-6 (U1102) and (GS) gp112 and identification of the Z29-specified homolog. Virology 1992;191:511-6.
28. Ellinger K, Neipel F, Foa Tomasi L, Campadelli Fiume G, Fleckenstein B. The glycoprotein B homologue of human herpesvirus 6. J Gen Virol 1993;74:495-500.
29. Campadelli Fiume G, Guerrini S, Liu X, Foà Tomasi L. Monoclonal antibodies to glycoprotein B differentiate human herpesvirus 6 into two clusters, variants A and B. J Gen Virol 1993;74:2257-62.
30. Balachandran N, Amelse RE, Zhou WW, Chang CK. Identification of proteins specific for human herpesvirus 6-infected human T cells. J Virol 1989;63:2835-40.
31. Foà-Tomasi L, Boscaro A, di Gaeta S, Campadelli Fiume G. Monoclonal antibodies to gp100 inhibit penetration of human herpesvirus 6 and polykaryocyte formation in susceptible cells. J Virol 1991;65:4124-9.
32. Liu DX, Gompels UA, Foà Tomasi L, Campadelli Fiume G. Human herpesvirus-6 glycoprotein H and L homologs are components of the gp100 complex and the gH external domain is the target for neutralizing monoclonal antibodies. Virology 1993;197:12-22.
33. Cirone M, Campadelli Fiume G, Foà-Tomasi L, Torrisi MR, Faggioni A. Human herpesvirus 6 envelope glycoproteins B and H-L complex are undetectable on the plasma membrane of infected lymphocytes. AIDS Res Hum Retroviruses 1994;10:175-9.

34. Schirmer EC, Wyatt LS, Yamanishi K, Rodriguez WJ, Frenkel N. Differentiation between two distinct classes of viruses now classified as human herpesvirus 6. Proc Natl Acad Sci U S A 1991;88:5922-6.
35. Ablashi DV, Balachandran N, Josephs SF, Hung CL, Krueger GR, Kramarsky B, et al. Genomic polymorphism, growth properties, and immunologic variations in human herpesvirus-6 isolates. Virology 1991;184:545-52.
36. Aubin JT, Collandre H, Candotti D, Ingrand D, Rouzioux C, Burgard M, et al. Several groups among human herpesvirus 6 strains can be distinguished by Southern blotting and polymerase chain reaction. J Clin Microbiol 1991;29:367-72.
37. Mirandola P, Menegazzi P, Merighi S, Ravaioli T, Cassai E, Di Luca D. Temporal mapping of transcripts in herpesvirus 6 variants. J Virol 1998;72:3837-44.
38. Ablashi D, Agut H, Berneman Z, Campadelli Fiume G, Carrigan D, Ceccherini Nelli L, et al. Human herpesvirus-6 strain groups: a nomenclature. Arch Virol 1993;129:363-6.
39. Ranger S, Patillaud S, Denis F, Himmich A, Sangare A, M'Boup S, et al. Seroepidemiology of human herpesvirus-6 in pregnant women from different parts of the world. J Med Virol 1991;34:194-8.
40. Adams O, Krempe C, Kogler G, Wernet P, Scheid A. Congenital infections with human herpesvirus 6. J Infect Dis 1998;178:544-6.
41. Pruksananonda P, Hall CB, Insel RA, McIntyre K, Pellett PE, Long CE, et al. Primary human herpesvirus 6 infection in young children. N Engl J Med 1992;326:1445-50.
42. Hall CB, Long CE, Schnabel KC, Caserta MT, McIntyre KM, Costanzo MA, et al. Human herpesvirus-6 infection in children. A prospective study of complications and reactivation. N Engl J Med 1994;331:432-8.
43. Kasolo FC, Mpabalwani E, Gompels UA. Infection with AIDS-related herpesviruses in human immunodeficiency virus-negative infants and endemic childhood Kaposi's sarcoma in Africa. J Gen Virol 1997;78:847-55.
44. Portolani M, Cermelli C, Moroni A, Bertolani MF, Di Luca D, Cassai E, et al. Human herpesvirus-6 infections in infants admitted to hospital. J Med Virol 1993;39:146-51.
45. Tanaka K, Kondo T, Torigoe S, Okada S, Mukai T, Yamanishi K. Human herpesvirus 7: another causal agent for roseola (exanthem subitum). J Pediatr 1994;125:1-5.
46. Caserta MT, Hall CB, Schnabel K, Long CE, D'Heron N. Primary human herpesvirus 7 infection: a comparison of human herpesvirus 7 and human herpesvirus 6 infections in children. J Pediatr 1998;133:386-9.
47. Di Luca D, Mirandola P, Ravaioli T, Bigoni B, Cassai E. Distribution of HHV-6 variants in human tissues. Infectious Agents and Disease 1996;5:203-14.
48. Leach CT, Newton ER, McParlin S, Jenson HB. Human herpesvirus 6 infection of the female genital tract. J Infect Dis 1994;169:1281-3.
49. Okuno T, Oishi H, Hayashi K, Nonogaki M, Tanaka K, Yamanishi K. Human herpesviruses 6 and 7 in cervixes of pregnant women. J Clin Microbiol 1995;33:1968-70.
50. Dunne WM Jr, Jevon M. Examination of human breast milk for evidence of human herpesvirus 6 by polymerase chain reaction. J Infect Dis 1993;168:250.
51. Daibata M, Taguchi T, Kamioka M, Kubonishi I, Taguchi H, Miyoshi I. Identification of integrated human herpesvirus 6 DNA in early pre-B cell acute lymphoblastic leukemia. Leukemia 1998;12:1002-4.
52. Luppi M, Barozzi P, Morris CM, Merelli E, Torelli G. Integration of human herpesvirus 6 genome in human chromosomes. Lancet 1998;352:1707-8.
53. Dewhurst S, McIntyre K, Schnabel K, Hall CB. Human herpesvirus 6 (HHV-6) variant B accounts for the majority of symptomatic primary HHV-6 infections in a population of U.S. infants. J Clin Microbiol 1993;31:416-8.
54. van Loon NM, Gummuluru S, Sherwood DJ, Marentes R, Hall CB, Dewhurst S. Direct sequence analysis of human herpesvirus 6 (HHV-6) sequences from infants and comparison of HHV-6 sequences from mother/infant pairs. Clin Infect Dis 1995;21:1017-9.
55. Kondo K, Kondo T, Okuno T, Takahashi M, Yamanishi K. Latent human herpesvirus 6 infection of human monocytes/macrophages. J Gen Virol 1991;72:1401-8.
56. Luppi M, Barozzi P, Morris C, Maiorana A, Garber R, Bonacorsi G, et al. Human herpesvirus 6 latently infects early bone marrow pregenitors in vivo. J Virol 1999;73:754-9.
57. DesJardin JA, Gibbons L, Cho E, Supran SE, Falagas ME, Werner BG, et al. Human herpesvirus 6 reactivation is associated with cytomegalovirus infection and syndromes in kidney transplant recipients at risk for primary cytomegalovirus infection. J Infect Dis 1998;178:1783-6.
58. Ratnamohan VM, Chapman J, Howse H, Bovington K, Robertson P, Byth K, et al. Cytomegalovirus and human herpesvirus 6 both cause viral disease after renal transplantation. Transplantation 1998;66:877-82.
59. Osman HK, Peiris JS, Taylor CE, Warwicker P, Jarrett RF, Madeley CR. "Cytomegalovirus disease" in renal allograft recipients: is human herpesvirus 7 a co-factor for disease progression? J Med Virol 1996;48:295-301.
60. Yalcin S, Karpuzoglu T, Suleymanlar G, Mutlu G, Mukai T, Yamamoto T, et al. Human herpesvirus 6 and human herpesvirus 7 infections in renal transplant recipients and healthy adults in Turkey. Arch Virol 1994;136:183-90.
61. Drobyski WR, Knox KK, Majewski D, Carrigan DR. Brief report: fatal encephalitis due to variant B human herpesvirus-6 infection in a bone marrow-transplant recipient. N Engl J Med 1994;330:1356-60.
62. Cone RW, Huang ML, Hackman RC, Corey L. Coinfection with human herpesvirus 6 variants A and B in lung tissue. J Clin Microbiol 1996;34:877-81.
63. Bosi A, Zazzi M, Amantini A, Cellerini M, Vannucchi AM, De Milito A, et al. Fatal herpesvirus 6 encephalitis after unrelated bone marrow. Bone Marrow Transplant 1998;22:285-8.
64. Secchiero P, Carrigan DR, Asano Y, Benedetti L, Crowley RW, Komaroff AL, et al. Detection of human herpesvirus 6 in plasma of children with primary infection and immunosuppressed patients by polymerase chain reaction. J Infect Dis 1995;171:273-80.
65. Clark DA, Ait Khaled M, Wheeler AC, Kidd IM, McLaughlin JE, Johnson MA, et al. Quantification of human herpesvirus 6 in immunocompetent persons and post-mortem tissues from AIDS patients by PCR. J Gen Virol 1996;77:2271-5.

66. Lusso P, Gallo RC. HHV-6 and CMV pneumonitis in immunocompromised patients. Lancet 1994;343:1647-8.
67. Knox KK, Carrigan DR. Disseminated active HHV-6 infections in patients with AIDS. Lancet 1994;343:577-8.
68. Lusso P, Gallo RC. Human herpesvirus 6 in AIDS. Immunol Today 1995;16:67-71.
69. Dolcetti R, Di Luca D, Carbone A, Mirandola P, De Vita S, Vaccher E, et al. Human herpesvirus 6 in human immunodeficiency virus-infected individuals: association with early histologic phases of lymphadenopathy syndrome but not with malignant lymphoproliferative disorders. J Med Virol 1996;48:344-53.
70. Akashi K, Eizuru Y, Sumiyoshi Y, Minematsu T, Hara S, Harada M, et al. Brief report: severe infectious mononucleosis-like syndrome and primary human herpesvirus 6 infection in an adult. N Engl J Med 1993;329:168-71.
71. Kempf W, Adams V, Wey N, Moos R, Schmid M, Avitabile E, et al. CD68+ cells of monocyte/macrophage lineage in the environment of AIDS-associated and classic-sporadic Kaposi sarcoma are singly or doubly infected with human herpesviruses 7 and 6B. Proc Natl Acad Sci U S A 1997;94:7600-5.
72. Fox JD, Briggs M, Ward PA, Tedder RS. Human herpesvirus 6 in salivary glands. Lancet 1990;336:590-3.
73. Lusso P, Gallo RC, DeRocco SE, Markham PD. CD4 is not the membrane receptor for HHV-6. Lancet 1989;1:730.
74. Cermelli C, Concari M, Carubbi F, Fabio G, Sabbatini AM, Pecorari M, et al. Growth of human herpesvirus 6 in HEPG2 cells. Virus Res 1996;45:75-85.
75. Levy JA, Ferro F, Lennette ET, Oshiro L, Poulin L. Characterization of a new strain of HHV-6 (HHV-6SF) recovered from the saliva of an HIV-infected individual. Virology 1990;178:113-21.
76. Ablashi DV, Lusso P, Hung CL, Salahuddin SZ, Josephs SF, Llana T, et al. Utilization of human hematopoietic cell lines for the propagation and characterization of HBLV (human herpesvirus 6). Dev Biol Stand 1989;70:139-46.
77. Albright AV, Lavi E, Black JB, Goldberg S, O'Connor MJ, Gonzalez-Scarano F. The effect of human herpesvirus-6 (HHV-6) on cultured human neural cells: oligodendrocytes and microglia. J Neurovirol 1998;4:486-94.
78. Challoner PB, Smith KT, Parker JD, MacLeod DL, Coulter SN, Rose TM, et al. Plaque-associated expression of human herpesvirus 6 in multiple sclerosis. Proc Natl Acad Sci U S A 1995;92:7440-4.
79. Knox KK, Harrington DP, Carrigan DR. Fulminant human herpesvirus six encephalitis in a human immunodeficiency virus-infected infant. J Med Virol 1995;45:288-92.
80. Luppi M, Barozzi P, Maiorana A, Marasca R, Torelli G. Human herpesvirus 6 infection in normal human brain tissue. J Infect Dis 1994;169:943-4.
81. Mackenzie IR, Carrigan DR, Wiley CA. Chronic myelopathy associated with human herpesvirus-6. Neurology 1995;45:2015-7.
82. Carrigan DR, Harrington D, Knox KK. Subacute leukoencephalitis caused by CNS infection with human herpesvirus-6 manifesting as acute multiple sclerosis. Neurology 1996;47:145-8.
83. Hall CB, Caserta MT, Schnabel KC, Long C, Epstein LG, Insel RA, et al. Persistence of human herpesvirus 6 according to site and variant: possible greater neurotropism of variant A. Clin Infect Dis 1998;26:132-7.
84. Sola P, Merelli E, Marasca R, Poggi M, Luppi M, Montorsi M, et al. Human herpesvirus 6 and multiple sclerosis: survey of anti-HHV-6 antibodies by immunofluorescence analysis and of viral sequences by polymerase chain reaction. J Neurol Neurosurg Psychiatry 1993;56:917-9.
85. Coates AR, Bell J. HHV-6 and multiple sclerosis. Nat Med 1998;4:537-8.
86. Soldan SS, Berti R, Salem N, Secchiero P, Flamand L, Calabresi PA, et al. Association of human herpes virus 6 (HHV-6) with multiple sclerosis: increased IgM response to HHV-6 early antigen and detection of serum HHV-6 DNA. Nat Med 1997;3:1394-7.
87. Wilborn F, Schmidt CA, Brinkmann V, Jendroska K, Oettle H, Siegert W. A potential role for human herpesvirus type 6 in nervous system disease. J Neuroimmunol 1994;49:213-4.
88. Sanders VJ, Felisan S, Waddell A, Tourtellotte WW. Detection of herpesviridae in postmortem multiple sclerosis brain tissue and controls by polymerase chain reaction. J Neurovirol 1996;2:249-58.
89. Martin C, Enbom M, Soderstrom M, Fredrikson S, Dahl H, Lycke J, et al. Absence of seven human herpesviruses, including HHV-6, by polymerase chain reaction in CSF and blood from patients with multiple sclerosis and optic neuritis. Acta Neurol Scand 1997;95:280-3.
90. Fillet AM, Lozeron P, Agut H, Lyon-Caen O, Liblau R. HHV-6 and multiple sclerosis. Nat Med 1998;4:537 [discussion 538].
91. Mirandola P, Stefan A, Brambilla E, Campadelli-Fiume G, Grimaldi LME. Absence of human herpesvirus 6 and 7 from spinal fluid and serum of multiple sclerosis patients. Neurology. In Press 1999.
92. Chang Y, Cesarman E, Pessin MS, Lee F, Culpepper J, Knowles DM, et al. Identification of herpesvirus-like DNA sequences in AIDS-associated Kaposi's sarcoma. Science 1994;266:1865-9.
93. Moore PS, Chang Y. Detection of herpesvirus-like DNA sequences in Kaposi's sarcoma in patients with and without HIV infection. N Engl J Med 1995;332:1181-5.
94. Kempf W, Adams V, Mirandola P, Menotti L, Di Luca D, Wey N, et al. Persistence of human herpesvirus 7 in normal tissues detected by expression of a structural antigen. J Infect Dis 1998;178:841-5.
95. Bovenzi P, Mirandola P, Secchiero P, Strumia R, Cassai E, Di Luca D. Human herpesvirus 6 (variant A) in Kaposi's sarcoma. Lancet 1993;341:1288-9.
96. Kempf W, Adams V, Pfaltz M, Briner J, Schmid M, Moos R, et al. Human herpesvirus type 6 and cytomegalovirus in AIDS-associated Kaposi's sarcoma: no evidence for an etiological association. Hum Pathol 1995;26:914-9.
97. Kashanchi F, Araujo J, Doniger J, Muralidhar S, Hoch R, Khleif S, et al. Human herpesvirus 6 (HHV-6) ORF-1 transactivating gene exhibits malignant transforming activity and its protein binds to p53. Oncogene 1997;14:359-67.

98. Thompson J, Choudhury S, Kashanchi F, Doniger J, Berneman Z, Frenkel N, et al. A transforming fragment within the direct repeat region of human herpesvirus type 6 that transactivates HIV-1. Oncogene 1994;9:1167-75.
99. Razzaque A. Oncogenic potential of human herpesvirus-6 DNA. Oncogene 1990;5:1365-70.
100. Patnaik M, Komaroff AL, Conley E, Ojo Amaize EA, Peter JB. Prevalence of IgM antibodies to human herpesvirus 6 early antigen (p41/38) in patients with chronic fatigue syndrome. J Infect Dis 1995;172:1364-7.
101. Di Luca D, Zorzenon M, Mirandola P, Colle R, Botta GA, Cassai E. Human herpesvirus 6 and human herpesvirus 7 in chronic fatigue syndrome. J Clin Microbiol 1995;33:1660-1.
102. Yalcin S, Kuratsune H, Yamaguchi K, Kitani T, Yamanishi K. Prevalence of human herpesvirus 6 variants A and B in patients with chronic fatigue syndrome. Microbiol Immunol 1994;38:587-90.
103. Ablashi DV, Josephs SF, Buchbinder A, Hellman K, Nakamura S, Llana T, et al. Human B-lymphotropic virus (human herpesvirus-6). J Virol Methods 1988;21:29-48.
104. Menotti L, Mirandola P, Locati M, Campadelli Fiume G. Trafficking to the plasma membrane of the seven-transmembrane protein encoded by human herpesvirus-6 U51 gene involves a cell-specific function present in T-lymphocytes. J Virol 1999;73:325-33.
105. Milne RSB, Nicholson L, Devaraj P, Gompels UA. The human herpesvirus 6 U51-encoded chemokine receptor-like protein down regulates expression of the chemokine RANTES. In: Proceedings of the 23rd International Herpesvirus Workshop; 1998; York, United Kingdom.

## References

[PDF_01289] Ablashi D. V., Josephs S. F., Buchbinder A., Hellman K., Nakamura S., Llana T., Lusso P., Kaplan M., Dahlberg J., Memon S. (1988). Human B-lymphotropic virus (human herpesvirus-6).. J Virol Methods.

[PDF_01160] Akashi K., Eizuru Y., Sumiyoshi Y., Minematsu T., Hara S., Harada M., Kikuchi M., Niho Y., Minamishima Y. (1993). Brief report: severe infectious mononucleosis-like syndrome and primary human herpesvirus 6 infection in an adult.. N Engl J Med.

[PDF_01005] Balachandran N., Amelse R. E., Zhou W. W., Chang C. K. (1989). Identification of proteins specific for human herpesvirus 6-infected human T cells.. J Virol.

[PDF_01132] Bosi A., Zazzi M., Amantini A., Cellerini M., Vannucchi A. M., De Milito A., Guidi S., Saccardi R., Lombardini L., Laszlo D. (1998). Fatal herpesvirus 6 encephalitis after unrelated bone marrow transplant.. Bone Marrow Transplant.

[PDF_00937] Braun D. K., Dominguez G., Pellett P. E. (1997). Human herpesvirus 6.. Clin Microbiol Rev.

[PDF_01001] Campadelli-Fiume G., Guerrini S., Liu X., Foà-Tomasi L. (1993). Monoclonal antibodies to glycoprotein B differentiate human herpesvirus 6 into two clusters, variants A and B.. J Gen Virol.

[PDF_01175] Cermelli C., Concari M., Carubbi F., Fabio G., Sabbatini A. M., Pecorari M., Pietrosemoli P., Meacci M., Guicciardi E., Carulli N. (1996). Growth of human herpesvirus 6 in HEPG2 cells.. Virus Res.

[PDF_01244] Chang Y., Cesarman E., Pessin M. S., Lee F., Culpepper J., Knowles D. M., Moore P. S. (1994). Identification of herpesvirus-like DNA sequences in AIDS-associated Kaposi's sarcoma.. Science.

[PDF_01017] Cirone M., Campadelli-Fiume G., Foà-Tomasi L., Torrisi M. R., Faggioni A. (1994). Human herpesvirus 6 envelope glycoproteins B and H-L complex are undetectable on the plasma membrane of infected lymphocytes.. AIDS Res Hum Retroviruses.

[PDF_01129] Cone R. W., Huang M. L., Hackman R. C., Corey L. (1996). Coinfection with human herpesvirus 6 variants A and B in lung tissue.. J Clin Microbiol.

[PDF_01107] DesJardin J. A., Gibbons L., Cho E., Supran S. E., Falagas M. E., Werner B. G., Snydman D. R. (1998). Human herpesvirus 6 reactivation is associated with cytomegalovirus infection and syndromes in kidney transplant recipients at risk for primary cytomegalovirus infection.. J Infect Dis.

[PDF_01072] Di Luca D., Mirandola P., Ravaioli T., Bigoni B., Cassai E. (1996). Distribution of HHV-6 variants in human tissues.. Infect Agents Dis.

[PDF_01281] Di Luca D., Zorzenon M., Mirandola P., Colle R., Botta G. A., Cassai E. (1995). Human herpesvirus 6 and human herpesvirus 7 in chronic fatigue syndrome.. J Clin Microbiol.

[PDF_01154] Dolcetti R., Di Luca D., Carbone A., Mirandola P., De Vita S., Vaccher E., Sighinolfi L., Gloghini A., Tirelli U., Cassai E. (1996). Human herpesvirus 6 in human immunodeficiency virus-infected individuals: association with early histologic phases of lymphadenopathy syndrome but not with malignant lymphoproliferative disorders.. J Med Virol.

[PDF_01081] Dunne W. M., Jevon M. (1993). Examination of human breast milk for evidence of human herpesvirus 6 by polymerase chain reaction.. J Infect Dis.

[PDF_01008] Foà-Tomasi L., Boscaro A., di Gaeta S., Campadelli-Fiume G. (1991). Monoclonal antibodies to gp100 inhibit penetration of human herpesvirus 6 and polykaryocyte formation in susceptible cells.. J Virol.

[PDF_00939] Gompels U. A., Nicholas J., Lawrence G., Jones M., Thomson B. J., Martin M. E., Efstathiou S., Craxton M., Macaulay H. A. (1995). The DNA sequence of human herpesvirus-6: structure, coding content, and genome evolution.. Virology.

[PDF_01208] Hall C. B., Caserta M. T., Schnabel K. C., Long C., Epstein L. G., Insel R. A., Dewhurst S. (1998). Persistence of human herpesvirus 6 according to site and variant: possible greater neurotropism of variant A.. Clin Infect Dis.

[PDF_00963] Inoue N., Dambaugh T. R., Rapp J. C., Pellett P. E. (1994). Alphaherpesvirus origin-binding protein homolog encoded by human herpesvirus 6B, a betaherpesvirus, binds to nucleotide sequences that are similar to ori regions of alphaherpesviruses.. J Virol.

[PDF_00990] Isegawa Y., Ping Z., Nakano K., Sugimoto N., Yamanishi K. (1998). Human herpesvirus 6 open reading frame U12 encodes a functional beta-chemokine receptor.. J Virol.

[PDF_01263] Kashanchi F., Araujo J., Doniger J., Muralidhar S., Hoch R., Khleif S., Mendelson E., Thompson J., Azumi N., Brady J. N. (1997). Human herpesvirus 6 (HHV-6) ORF-1 transactivating gene exhibits malignant transforming activity and its protein binds to p53.. Oncogene.

[PDF_01058] Kasolo F. C., Mpabalwani E., Gompels U. A. (1997). Infection with AIDS-related herpesviruses in human immunodeficiency virus-negative infants and endemic childhood Kaposi's sarcoma in Africa.. J Gen Virol.

[PDF_00934] Katsafanas G. C., Schirmer E. C., Wyatt L. S., Frenkel N. (1996). In vitro activation of human herpesviruses 6 and 7 from latency.. Proc Natl Acad Sci U S A.

[PDF_01251] Kempf W., Adams V., Mirandola P., Menotti L., Di Luca D., Wey N., Müller B., Campadelli-Fiume G. (1998). Persistence of human herpesvirus 7 in normal tissues detected by expression of a structural antigen.. J Infect Dis.

[PDF_01165] Kempf W., Adams V., Wey N., Moos R., Schmid M., Avitabile E., Campadelli-Fiume G. (1997). CD68+ cells of monocyte/macrophage lineage in the environment of AIDS-associated and classic-sporadic Kaposi sarcoma are singly or doubly infected with human herpesviruses 7 and 6B.. Proc Natl Acad Sci U S A.

[PDF_01194] Knox K. K., Harrington D. P., Carrigan D. R. (1995). Fulminant human herpesvirus six encephalitis in a human immunodeficiency virus-infected infant.. J Med Virol.

[PDF_01100] Kondo K., Kondo T., Okuno T., Takahashi M., Yamanishi K. (1991). Latent human herpesvirus 6 infection of human monocytes/macrophages.. J Gen Virol.

[PDF_01198] Luppi M., Barozzi P., Maiorana A., Marasca R., Torelli G. (1994). Human herpesvirus 6 infection in normal human brain tissue.. J Infect Dis.

[PDF_01103] Luppi M., Barozzi P., Morris C., Maiorana A., Garber R., Bonacorsi G., Donelli A., Marasca R., Tabilio A., Torelli G. (1999). Human herpesvirus 6 latently infects early bone marrow progenitors in vivo.. J Virol.

[PDF_01248] Moore P. S., Chang Y. (1995). Detection of herpesvirus-like DNA sequences in Kaposi's sarcoma in patients with and without HIV infection.. N Engl J Med.

[PDF_00959] Mori Y., Yagi H., Shimamoto T., Isegawa Y., Sunagawa T., Inagi R., Kondo K., Tano Y., Yamanishi K. (1998). Analysis of human herpesvirus 6 U3 gene, which is a positional homolog of human cytomegalovirus UL 24 gene.. Virology.

[PDF_00972] Neipel F., Ellinger K., Fleckenstein B. (1992). Gene for the major antigenic structural protein (p100) of human herpesvirus 6.. J Virol.

[PDF_00943] Nicholas J. (1996). Determination and analysis of the complete nucleotide sequence of human herpesvirus.. J Virol.

[PDF_01078] Okuno T., Oishi H., Hayashi K., Nonogaki M., Tanaka K., Yamanishi K. (1995). Human herpesviruses 6 and 7 in cervixes of pregnant women.. J Clin Microbiol.

[PDF_01117] Osman H. K., Peiris J. S., Taylor C. E., Warwicker P., Jarrett R. F., Madeley C. R. (1996). "Cytomegalovirus disease" in renal allograft recipients: is human herpesvirus 7 a co-factor for disease progression?. J Med Virol.

[PDF_01276] Patnaik M., Komaroff A. L., Conley E., Ojo-Amaize E. A., Peter J. B. (1995). Prevalence of IgM antibodies to human herpesvirus 6 early antigen (p41/38) in patients with chronic fatigue syndrome.. J Infect Dis.

[PDF_00985] Rotola A., Ravaioli T., Gonelli A., Dewhurst S., Cassai E., Di Luca D. (1998). U94 of human herpesvirus 6 is expressed in latently infected peripheral blood mononuclear cells and blocks viral gene expression in transformed lymphocytes in culture.. Proc Natl Acad Sci U S A.

[PDF_01024] Schirmer E. C., Wyatt L. S., Yamanishi K., Rodriguez W. J., Frenkel N. (1991). Differentiation between two distinct classes of viruses now classified as human herpesvirus 6.. Proc Natl Acad Sci U S A.

[PDF_01136] Secchiero P., Carrigan D. R., Asano Y., Benedetti L., Crowley R. W., Komaroff A. L., Gallo R. C., Lusso P. (1995). Detection of human herpesvirus 6 in plasma of children with primary infection and immunosuppressed patients by polymerase chain reaction.. J Infect Dis.

[PDF_00914] Takahashi K., Sonoda S., Higashi K., Kondo T., Takahashi H., Takahashi M., Yamanishi K. (1989). Predominant CD4 T-lymphocyte tropism of human herpesvirus 6-related virus.. J Virol.

[PDF_00954] Takeda K., Nakagawa N., Yamamoto T., Inagi R., Kawanishi K., Isegawa Y., Yamanishi K. (1996). Prokaryotic expression of an immediate-early gene of human herpesvirus 6 and analysis of its viral antigen expression in human cells.. Virus Res.

[PDF_00968] Tigue N. J., Matharu P. J., Roberts N. A., Mills J. S., Kay J., Jupp R. (1996). Cloning, expression and characterization of the proteinase from human herpesvirus 6.. J Virol.

[PDF_00975] Yamamoto M., Black J. B., Stewart J. A., Lopez C., Pellett P. E. (1990). Identification of a nucleocapsid protein as a specific serological marker of human herpesvirus 6 infection.. J Clin Microbiol.

[PDF_01095] van Loon N. M., Gummuluru S., Sherwood D. J., Marentes R., Hall C. B., Dewhurst S. (1995). Direct sequence analysis of human herpesvirus 6 (HHV-6) sequences from infants and comparison of HHV-6 sequences from mother/infant pairs.. Clin Infect Dis.

